# A Flexible Interdigital Electrode Used in Skin Penetration Promotion and Evaluation with Electroporation and Reverse Iontophoresis Synergistically

**DOI:** 10.3390/s18051431

**Published:** 2018-05-04

**Authors:** Rongjian Zhao, Chenshuo Wang, Fei Lu, Lidong Du, Zhen Fang, Xiuhua Guo, Jen-Tsai Liu, Ching-Jung Chen, Zhan Zhao

**Affiliations:** 1 State Key Laboratory of Transducer Technology, Institute of Electronics, Chinese Academy of Sciences, Beijing 100080, China; zhaorongjian15@mails.ucas.ac.cn (R.Z.); wangchenshuo16@mails.ucas.ac.cn (C.W.); lufei15@mails.ucas.ac.cn (F.L.); lddu@mail.ie.ac.cn (L.D.); zfang@mail.ie.ac.cn (Z.F.); 2 University of Chinese Academy of Sciences, Beijing 100049, China; jtliu@ucas.edu.cn (J.-T.L.); cjchen@ucas.ac.cn (C.-J.C.); 3 School of Public Health, Capital Medical University, Beijing 100069, China; guoxiuh@ccmu.edu.cn; 4 Beijing Municipal Key Laboratory of Clinical Epidemiology, Beijing 100069, China

**Keywords:** flexible interdigital microelectrode, electroporation, reverse iontophoresis, promotion penetration, impedance

## Abstract

Skin penetration is related to efficiencies of drug delivery or ISF extraction. Normally, the macro-electrode is employed in skin permeability promotion and evaluation, which has the disadvantages of easily causing skin damage when using electroporation or reverse iontophoresis by alone; furthermore, it has large measurement error, low sensitivity, and difficulty in integration. To resolve these issues, this paper presents a flexible interdigital microelectrode for evaluating skin penetration by sensing impedance and a method of synergistical combination of electroporation and reverse iontophoresis to promote skin penetration. First, a flexible interdigital microelectrode was designed with a minimal configuration circuit of electroporation and reverse iontophoresis for future wearable application. Due to the variation of the skin impedance correlated with many factors, relative changes of it were recorded at the end of supply, different voltage, or constant current, times, and duration. It is found that the better results can be obtained by using electroporation for 5 min then reverse iontophoresis for 12 min. By synergistically using electroporation and reverse iontophoresis, the penetration of skin is promoted. The results tested in vivo suggest that the developed microelectrode can be applied to evaluate and promote the skin penetration and the designed method promises to leave the skin without damage. The electrode and the method may be beneficial for designing noninvasive glucose sensors.

## 1. Introduction

As traditional blood glucose testing is invasive, it brings great pain to the patient. However, diabetes patients need to measure glucose levels more than 8 times a day [1,2,3]. Thus, it also brings a big psychological burden to patients and could result in poor compliance. In recent years, researchers have used the ISF (interstitial-fluid) to replace blood for measuring human glucose concentration by a method of reverse iontophoresis extraction of ISF through the skin and achieved some results [4,5,6,7]. This method determines the blood glucose level based on monitoring the glucose concentration in ISF because a close relationship exists between the glucose concentration of the ISF and that of blood [8].

Due to the small volume of extracted ISF and the strong current density, reverse iontophoresis may damage skin. In addition, the measurement is inaccurate if the patient is moving, exercising, sweating, or experiencing rapid temperature changes [5,6]. When the extracted tissue fluid reaches a certain level, the glucose electrode can accurately test the results. As we know, the reverse iontophoresis applies a constant current to the skin. Under the action of the electric field, the positive and negative ions move in the subcutaneous tissue toward the cathode and anode respectively. The electric field of reverse iontophoresis forms a positive to negative ion current, which is used to carry the glucose in the subcutaneous tissue fluid to the skin surface [9]. The electroporation was firstly used for transdermal drug penetration, and it acts on the skin with a brief high-voltage pulse to achieve transient nanometer-wide aqueous pores in the formation of lipid bilayers in the skin, increasing the skin’s penetration during action [10]. There is a certain relationship between skin impedance and penetration. Under low frequency conditions (100 Hz~1000 Hz), skin impedance can better reflect its penetration. The smaller the impedance of skin becomes, the greater the penetration of skin becomes [11,12,13]. Traditionally, skin permeability evaluation, which is realized by impedance detected at a certain frequency based on macro-electrode, has the disadvantages of large measurement error, low sensitivity, and difficulty in integration [14,15]. Therefore, skin with a multilayer structure can be better characterized by its electrical properties using the interdigital microelectrode [16]. In the experiment, a low-cost flexible gold interdigital microelectrode is designed to evaluate skin penetration promotion by measuring skin impedance. The method of electroporation was used to open the water-containing channel to increase the penetration of the skin, and the reverse iontophoresis’s method was used to extract the glucose in the ISF. It is expected that the extraction time is shorter and current smaller, the extracted amount is larger. Synergistical combination of electroporation and reverse iontophoresis to achieve the expectation may be a way.

The objectives of this in vivo study are to evaluate and promote the skin penetration by interdigital microelectrode and to find suitable permeation-enhanced conditions through the designed method that can be used to guide ISF extraction, thereby providing guidance for blood glucose monitoring and diabetes management. In the study, different types of electroporation, various reverse iontophoresis and combination of electroporation and reverse iontophoresis for in vivo skin penetration experiments by interdigital microelectrode were applied and affected factors of these methods on skin penetration were explored, and noninvasive extracting glucose in the ISF was hoped for.

## 2. Materials and Methods

### 2.1. Fabrication of Flexible Gold Interdigital Microelectrode

**Figure 1:**
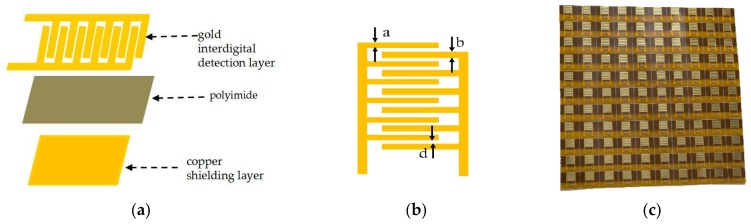
(**a**) Schematic of the flexible gold interdigital microelectrode multilayers; (**b**) The structure diagram of interdigital microelectrodes; (**c**) The fabricated multilayer microelectrode.

The skin penetration promotion and evaluation was conducted with the same electrodes, which is designed with the form of a flexible gold interdigital microelectrode. The interdigital structure is one of the most commonly used form. It is often applied to the detection of multi-layer material properties because it can sensitively detect changes in material properties [17]. In this study, the design of the electrode was performed according to the literature [18]. Figure 1a shows the schematic of the flexible gold interdigital microelectrode multilayers. The gold interdigital detection layer (0.2 µm) is located on the top of the electrode. The material of the middle layer (12.5 µm) is polyimide. A copper shielding layer (0.2 µm) is formed on the bottom of the electrode, and the area of the shielding layer completely covers the corresponding area of the gold interdigital detection layer to reduce external interference. Figure 1b is the draft of interdigital electrode structure. “a”, “b”, “d” represent the left finger width, the right finger width and electrode spacing, respectively. The sizes/geometries of the electrodes were tested and validated to suit the impedance test of different individuals and different skin parts [19]. The optimum finger width parameter of the flexible gold interdigital microelectrode is a = 100 µm, b = 300 µm, d = 135 µm. Figure 1c is the multilayer sensors manufactured by HLDtech, Inc. (Beijing, China) based on flexible FPC technology.

### 2.2. Circuit Design of the Electroporation

High voltage is used in electroporation, but the actual voltage on the skin is relatively low. Chizmadzhev Y et al. (1997) believe that combining with the theory and experiment, this voltage can choose the moderate value *U_skin_* = 10~60 V [20]. Therefore, the pulse voltage of electroporation was 0~60 V in this paper. The reduction of the voltage used for electroporation can greatly simplify the complexity of the circuit and enable miniaturization of the electroporation.

**Figure 2:**
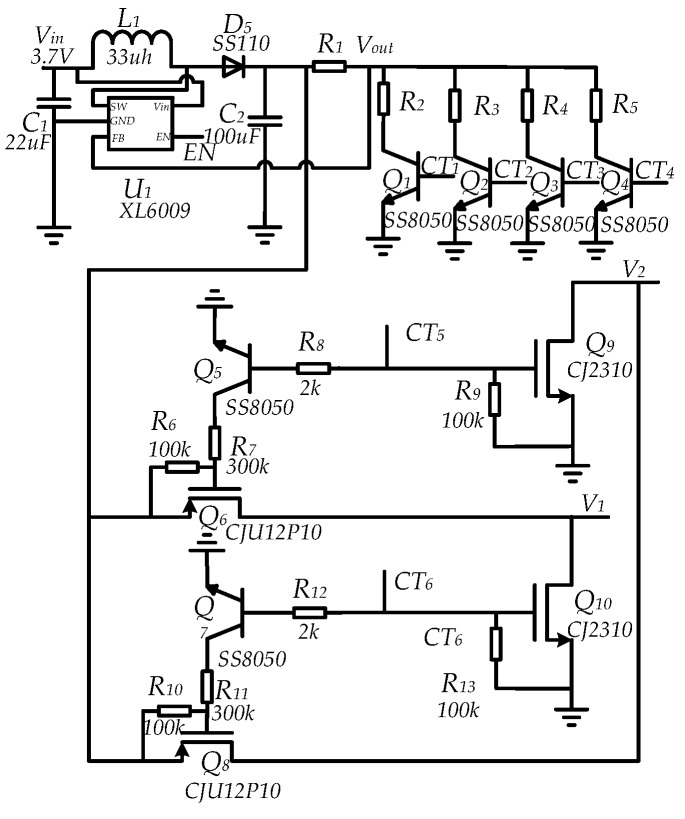
Circuit diagram of the electroporation for high-voltage electric pulse.

The circuit of electroporation consists of two parts, voltage regulation and output control circuit as shown in Figure 2. While the voltage regulation consists of an integrated circuit (*U*_1_), 2 capacitors (*C*_1_ to *C*_2_), 1 diodes (*D*_1_), a resistor (*R*_2_) and a series of variable resistors (*R*_2_ to *R*_5_) for an adjustable voltage. The *U*_1_ is a boost power supply module and it can boost a 0 V into 60 V. The output voltage is determined by the resistor *R*_2_~*R*_5_. *R_x_* is controlled by a switch. When *CT_x_* is high, the corresponding switch turns on the response resistor and four different voltages can be achieved.

The output voltage can be derived using Equation (1):

(1) $$Vout=1.25\times (1+R_{1}/Rx)$$

The combination of *CT*_5_ and *CT*_6_ controls can implement different types of pulses. When *CT*_5_ is high, *Q*_6_ and *Q*_9_ are on, and the positive pulse voltage is output. When *CT*_6_ is high, *Q*_8_ and *Q*_10_ are on, and the negative pulse voltage is output. When *CT*_5_ and *CT*_6_ are alternately high, a bidirectional pulse voltage is output.

### 2.3. Circuit Design of the Reverse Iontophoresis

**Figure 3:**
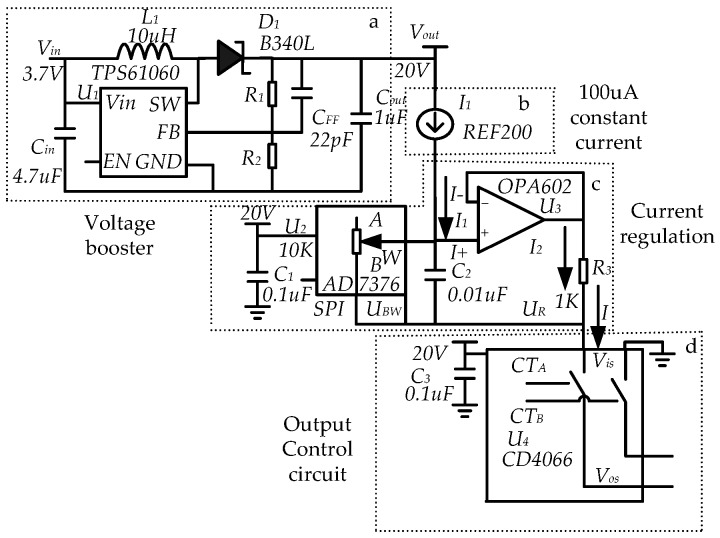
Circuit diagram of the reverse iontophoresis. (**a**) Voltage booster; (**b**) Constant current source; (**c**) Current regulation; (**d**) Output control circuit.

The schematic circuit diagram of reverse iontophoresis is shown in Figure 3. The circle can provide a variable current in the range of 100 μA~1.1 mA. The circuit is composed of four parts: voltage booster; 100 μA constant current; current regulation and output control circuit.

The current regulation of circuit is the core part of the circuit of reverse iontophoresis. It uses 100 μA constant current source through the operational amplifier and digitally controlled potentiometer to use the “virtual short” and “virtual-off” characteristics of the operational amplifier to achieve adjustable current output. The voltage *U_+_* and *U_−_* at the input operational amplifier (*U*_3_) are equal. The current passing operational amplifier (*U*_3_) is equal to 0 A.

(2) $$U_{+}=U-$$

(3) $$I_{+}=I-=0A$$

From Equations (2) and (3), the voltage *U_R_* across *R*_3_, and the voltage *U_BR_* across *R_BW_* are equal.

(4) $$U_{BW}=U_{R}=I_{1}\times R_{BW}=I_{2}\times R$$

Therefore,

(5) $$I_{2}=N\times I_{1}$$

where *I*_1_ and *I*_2_ are the current through *R_BW_* and *R*_3_, respectively.

Combining Equations (1) and (2), the total current *I* at the output can be expressed by Equation (6).

(6) $$I=(I_{1}+I_{2})=(N+1)\times I_{1}$$

In Equation (4), *R_BW_* is the access resistance of the AD7376 (Figure 3c), which is a high voltage digitally controlled potentiometer and whose range is 0~10 kΩ, so the total output current is 100 µA~1.1 mA.

### 2.4. Experiments on Skin Penetration In Vivo

**Figure 4:**
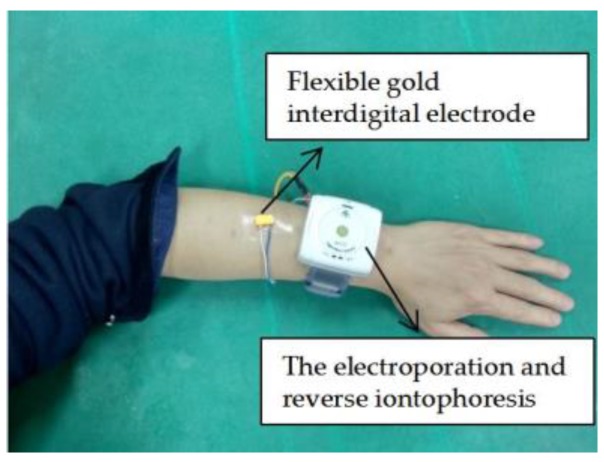
Pictures of skin penetration promotion test with the device of electroporation and reverse iontophoresis.

All experiments were conducted on skin of volunteers in vivo as shown in Figure 4. Different modes of electroporation and reverse iontophoresis were applied to the flexible gold interdigital electrode. Firstly, the effect of electroporation and reverse iontophoresis by measuring skin impedance was studied, then the optimal penetration-promoting experimental conditions were obtained. One-way analysis of variance was also used to determine whether there were significant differences between reverse iontophoresis parameters and the electroporation parameter for the test parameters of *Z_before_*/*Z_after_* (*f* = 100 Hz) with Potentiosta EIS of gamry Reference600 electrochemical workstation [21]. The test conditions were controlled at 25 °C room temperature, 40% relative humidity through the air conditioner. When the electrode attached the skin after 40 min of stable period, the skin will produce sweat to humidity saturation. Test parameter of *Z_before_*/*Z_after_* makes sense to avoid differences among various skins and test conditions. For the test parameter of *Z_before_*/*Z_after_*, if its value is greater than 1, it means that the post-experimental skin impedance is smaller than the pre-experimental skin impedance.

## 3. Results and Discussion

### 3.1. Evaluation of the Circuit of Electroporation and Reverse Iontophoresis

**Figure 5:**
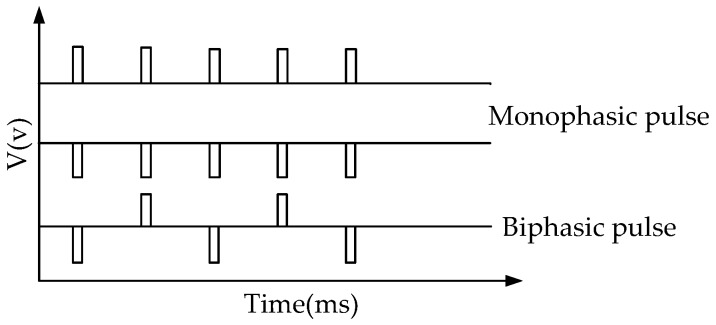
Output waveform of the micro electroporation’s circle.

The accuracy of the minimal configured electroporation and reverse iontophoresis circuit was evaluated by simulation test. The electroporation’s evaluation results are summarized in Figure 5 and Table 1 and Table 2. The load resistor of 100 k (VSMP2512100K, ±0.01%, EUREK, ShenZhen, China) is connected to the output by measuring waveform through a digital oscilloscope (MSO-X3054A, Agilent Technologies Inc., Santa Clara, CA, USA). Figure 5 shows the circle of electroporation being able to generate a pulsed electric waveform at various voltages with a specific pulse width. The circle is shown to be fully programmable configuration. Table 1 shows that the maximum voltage percentage error of electric pulses waveform is less than 3%. The percentage error of the circle on generating electric pulses with specific pulse width is found to be smaller than 1% and this means that the circle is accurate on generating electric pulse (Table 2).

**Figure 6:**
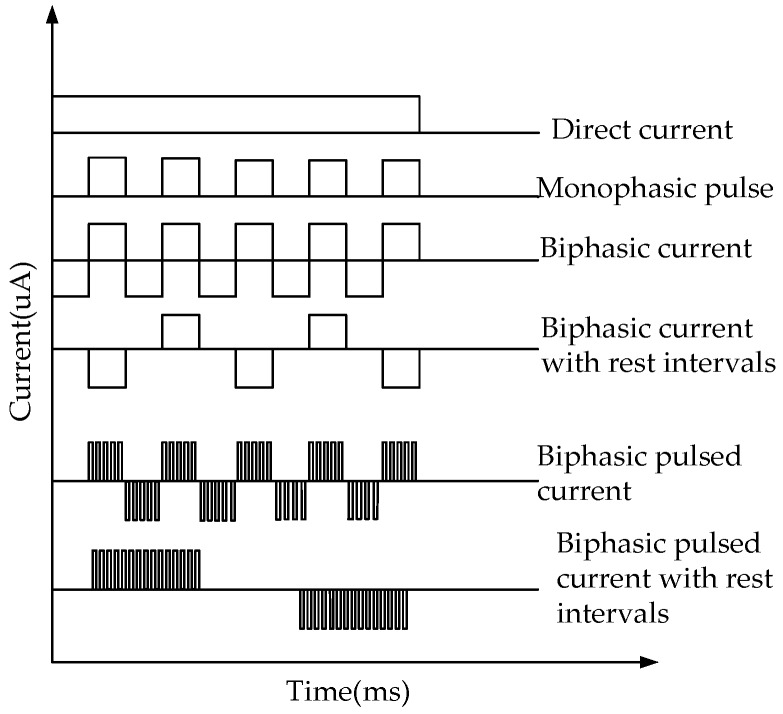
Output waveform of the reverse iontophoresis’s circle.

The constant current for different waveform in revers iontophoresis was evaluated. Two resistors with resistance of 10 k (VSMP080510K, ±0.01%, EUREK, Shenzhen, China) and 100 k (VSMP2512100K, ±0.01%, EUREK, Shenzhen, China) as circuit load were used to simulate the resistance of skin [22]. These resistors are connected to the output of reverse iontophoresis. A digital oscilloscope (MSO-X3054A, Agilent Technologies Inc., Santa Clara, CA, USA) was used to measure the potential difference across the resistors. The magnitude of the current flowing through the resistor can be calculated from Ohm’s Law. The evaluation results of the reverse iontophoresis are summarized in Figure 6 and Table 3. Figure 6 shows the circle of reverse iontophoresis being able to generate a various pulsed electric waveform. It was found that the accuracy of the reverse iontophoresis’s constant current was about ±2% and accuracy of the circle of reverse iontophoresis’s pulse and bipolar current error timing, were smaller than ±1.5% (see Table 3). The error on timing when generating pulsed and bipolar waveform of currents may partly come from the microprocessor because it has an accuracy limit of ±1% on timing.

### 3.2. Evaluation of Electroporation and Reverse Iontophoresis for Promoting Penetration of Skin by Measuring Skin Impedance In Vivo

The device is Li-battery-powered (3.7 V). If the average working current of the system is 50 mA, it can work 70 cycles (one cycle of 17 min) under the battery capacity of 1000 mA/h and extract more tissue fluid with electroporation and reverse iontophoresis synergistically.

**Figure 7:**
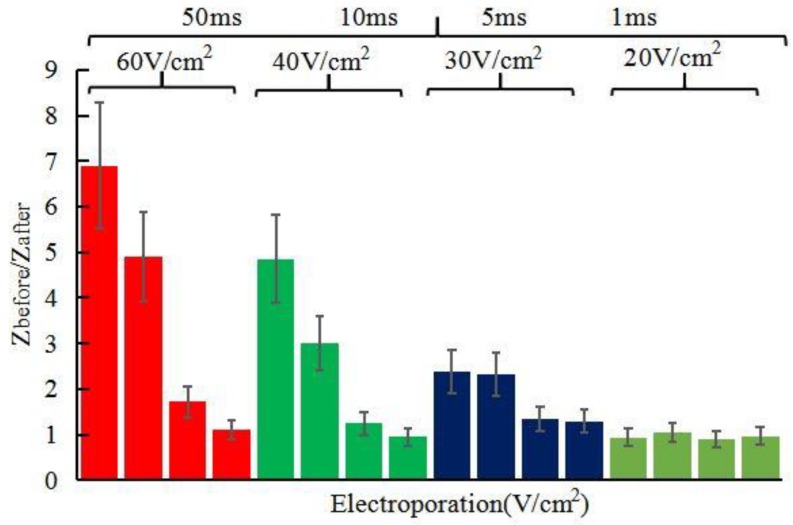
Effect of different pulse voltage and width pulsed-monophasic electric waveform of electroporation on the ratio of pre-experimental to post-experimental skin impedance (*Z_before_*/*Z_after_*). All skin impedance measurements were conducted at 100 Hz. For the electroporation setting, they all have the number of pulses per second of 10.

**Figure 8:**
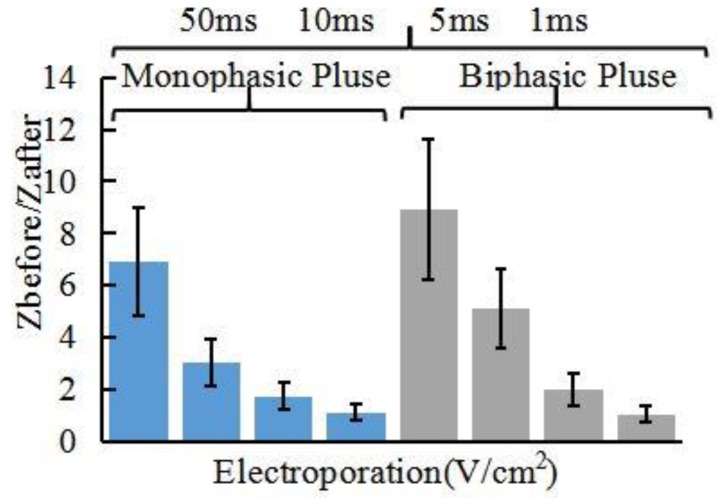
Effect of pulsed-monophasic and pulsed-biphasic electric waveform of electroporation on the ratio of pre-experimental to post-experimental skin impedance (*Z_before_*/*Z_after_*). All skin impedance measurements were conducted at 100 Hz. For the electroporation setting, they all have the number of pulses per second of 10 and the electric field strength of 40 V/cm^2^.

**Figure 9:**
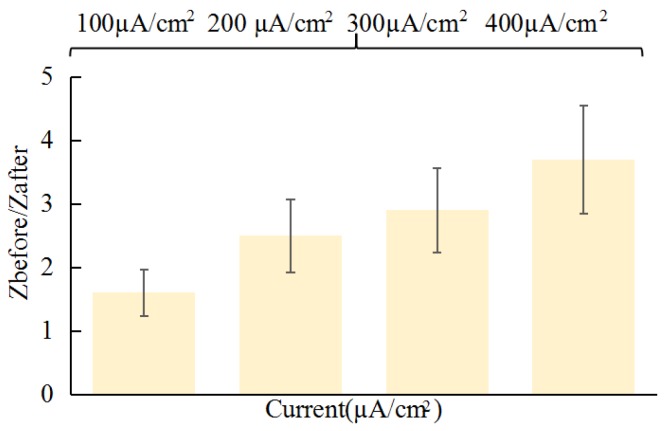
Effect of reverse iontophoresis (symmetricalbiphasic dc; different current density; reversed every 3 min during the 12 min) on the ratio of pre-experimental to post-experimental skin impedance (*Z_before_*/*Z_after_*).

**Figure 10:**
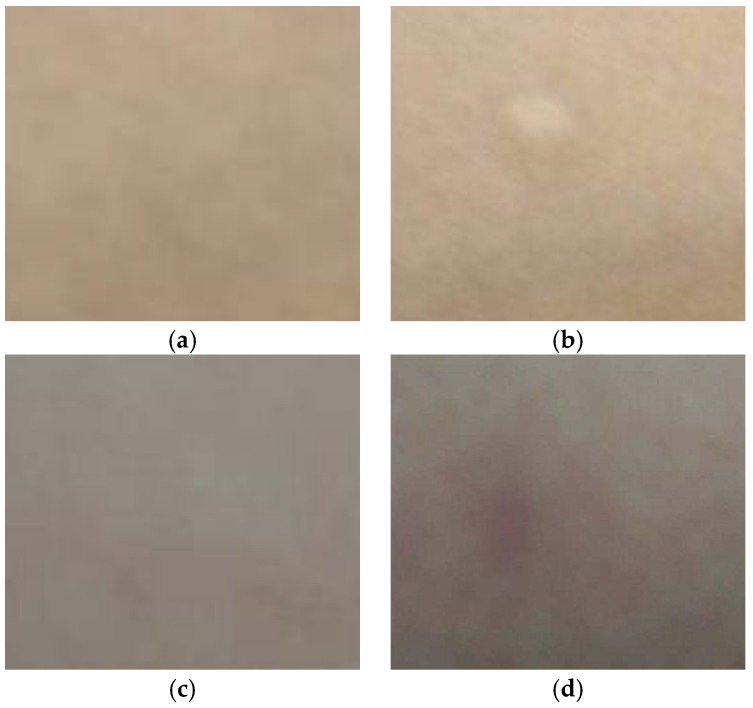
Pictures of the skin surface before and after each experiment. (**a**,**b**) Before and after experiment, respectively, with application of electroporation (pulse voltage density = 60 V/cm^2^, pulse width = 10 ms; number of pulses per second = 10); (**c**,**d**) Before and after experiment, with application of reverse iontophoresis (Symmetrical biphasic dc, current density = 0.3 mA/cm^2^; reversed every 3 min during the 12 min).

Our in vivo studies of promoting penetration of skin by electroporation, reverse iontophoresis and synergistical use of them are summarized in Figure 7, Figure 8, Figure 9 and Figure 10. Figure 7 shows the test results of the ratio of pre-experimental to post-experimental skin impedance under the condition of different pulse voltage (60~20 V/cm^2^), width (50~1 ms) of pulsed-monophasic electric waveform of electroporation. It was found that the increase of pulse voltage and width strength of electroporation could generally further decrease post experimental skin impedance under the same electroporation setting and this may be due to formation of nanochannels of ISF. The resulting drop in skin impedance is effectively proportional to the electrical field strength (pulse voltage × total pulse width), and this may be due to more or larger nanochannels being formed [16]. Figure 8 shows the effect of pulsed-monophasic and pulsed-biphasic electric waveform of electroporation on the ratio of pre-experimental to post-experimental skin impedance. It was observed that pulsed-biphasic electric waveform causes larger impedance changes than pulsed-monophasic electric waveform under the same conditions, which means that pulsed-biphasic electric waveform of electroporation has better promotion permeation efficiency than pulsed-monophasic waveform.

Figure 9 shows effect of reverse iontophoresis on the ratio of pre-experimental to post-experimental skin impedance. Under the same reverse iontophoresis setting, it was found that the increase of electrical field strength of reverse iontophoresis could generally further decrease post experimental skin impedance. Further, effect of reverse iontophoresis on the ratio of pre-experimental to post-experimental skin impedance decrease smaller than electroporation’s.

**Figure 9.** Effect of reverse iontophoresis (symmetricalbiphasic dc; different current density; reversed every 3 min during the 12 min) on the ratio of pre-experimental to post-experimental skin impedance (*Z_before_*/*Z_after_*).

From the above studies, electroporation can reduce skin impedance more than reverse iontophoresis that means electroporation can improve skin penetration more. Figure 10 shows the pictures of the skin surface before and after experiment. If electric field strength of electroporation continues to increase, it is not clinically feasible for safety reasons and induction of pain sensation and involuntary muscle contractions. The electric field strength (>40 V/cm^2^) and pulse width (>10 ms) of electroporation are uncomfortable to some users (Figure 10a,b). However, in practical applications, such as using reverse iontophoresis to extract glucose of tissue fluids, long-time and high-intensity currents can easily cause skin irritation (Figure 10c,d). When the current intensity is greater than 300 μA/cm^2^, the skin is prone to damage. So that the optimized condition of electroporation on the human body is electric field strength (<40 V/cm^2^) and pulse width (<10 ms) and the condition of reverse iontophoresis on the human body is less than 300 μA/cm^2^.

**Figure 11:**
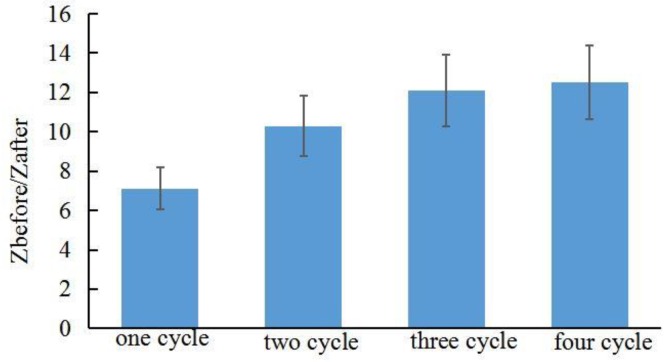
Effect of electroporation (electric field strength = 40 V/cm^2^; pulse width = 10 ms; number of pulses per second = 10, duration 5 min) and reverse iontophoresis (symmetrical biphasic dc; current density = 0.1 mA/cm^2^; reversed every 3 min during the 12 min) on the ratio of pre-experimental to post-experimental skin impedance at various times after the promotion penetration experiment.

Figure 11 shows the ratio of pre-experimental skin impedance to post-experimental skin impedance at various times after synergistically application of electroporation (electric field strength = 40 V/cm^2^, pulse width = 10 ms, number of pulses per second = 10, duration 5 min) and reverse iontophoresis (symmetrical biphasic dc; current density = 0.1 mA/cm^2^; reversed every 3 min during the 12 min). The combination of electroporation and reverse iontophoresis has additional benefits to promoting penetration. After one cycle of supplying the electroporation and revers iontophoresis, the impedance changes greatly before and after the experiment, which means that the skin has a strong penetration. The synergy of reverse iontophoresis and electroporation was more than doubled compared to the use of reverse iontophoresis alone. A steady decrease in the ratio with time was observed from more cycle penetration. Continuing to promote penetration, the value of *Z_before_*/*Z_after_* changes gradually become smaller and slowly. In the process of promoting penetration, the skin was not significantly damaged. Therefore, Skin penetration promotion with electroporation and reverse iontophoresis synergistically can hopefully be improved at a lower current density and relatively weak electric field strength without damaging the skin.

### 3.3. In Vivo Evaluation of Electroporation and Reverse Iontophoresis Synergistically for ISF Extraction by Weight

**Figure 12:**
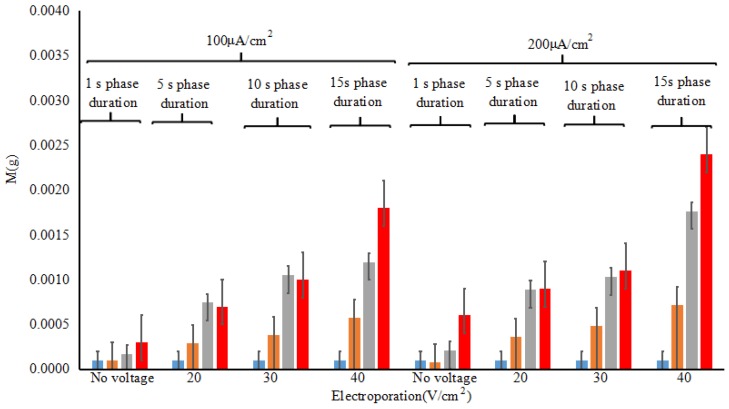
Effect of electroporation (electric field strength = 0~40 V/cm^2^, pulse width = 10 ms, number of pulses per second = 10, duration 5 min) and reverse iontophoresis (symmetrical biphasic dc; current density = 0.1~0.2 mA/cm^2^; reversed every 3 min during the 12 min) on the difference of ISF extraction pre-experimental to post-experimental by measuring weight under different conditions.

Under the safe conditions, the experiment of using electroporation and reverse iontophoresis synergistically to extract tissue fluid in vivo was carried out by standard ECG electrode. The extracted glucose is collected by a gel on the surface of the electrode. To accurately measure the amount of the extracted tissue fluid, the standard ECG electrodes are weighed separately before and after the electroporation and reverse iontophoresis experiment using precision electronic balance (ESJ182-4, precision 0.1 mg Shenyang Longteng Electronics Co., Ltd., Shenyang, China). Figure 12 shows the relationship of the weight of ISF extracted pre-experimental to post-experimental under different test conditions after synergistically application of electroporation (electric field strength = 40 V/cm^2^, pulse width = 10 ms, number of pulses per second = 10, duration 5 min) and reverse iontophoresis (symmetrical biphasic dc; current density = 0.1 mA/cm^2^; reversed every 3 min during the 12 min). At present, the glucose electrode can accurately measure the glucose concentration under the condition of 0.5 mg sample size. The amount of tissue fluid extracted by electroporation and reverse iontophoresis synergistically can reach up to 2.5 mg to meet blood glucose detection without harming human skin.

## 4. Conclusions

In this paper, a flexible interdigital microelectrode was designed for evaluating skin penetration by sensing impedance and a new method for noninvasive penetration promoting of skin with synergistical combination of electroporation and reverse iontophoresis has been demonstrated in vivo. First, a low-cost flexible gold interdigital microelectrode is designed to evaluate skin penetration promotion. Then, the battery-powered and programmable circuit of electroporation for high-electric pulse and reverse iontophoresis for a variable current is designed, which is suitable for wearable application in future. The electroporation can deliver precisely pulsed and pulsed-biphasic voltage with specific pulse width and number of pulses per second. Also, the reverse iontophore are precise when delivering a variable current in the range of 100 μA~1.1 mA with a various pulsed electric waveform. Using electroporation and reverse iontophoresis in vivo experiments, it is found that both electroporation and reverse iontophoresis can promote penetration of skin, but electroporation improves skin penetration more. If electric field strength of electroporation increase, the penetration of the skin increases correspondingly, but electric field strength of electroporation continues to increase that it is not clinically feasible for safety reasons. The reverse iontophoresis promotes the penetration of the skin relatively weakly, and the extraction of glucose and other tissue fluid target can be only extracted by reverse iontophoresis. The long-term, high-intensity current of reverse iontophoresis easily causes skin damage. Therefore, this method is expected to increase the penetration of the skin by interdigital microelectrode without harming the skin and is of great significance for the clinical application of noninvasive extracted tissue fluid for blood glucose measurement. The initial experiment was conducted only on a small group of people and the amount of tissue fluid extracted by electroporation and reverse iontophoresis synergistically can reach up to 2.5 mg to meet blood glucose detection. The selection of experimental conditions may only be suitable for this group of people. Future efforts will be concentrated on applying this method to large-scale populations in clinical applications and designed as a clinical wearable device to improve the amount of ISF for noninvasive blood glucose concentration detection.

## Figures and Tables

**Table 1:** Error estimation of the electroporation on generating electric pulses with specific pulse voltage.

Parameters		Load = 100 k, Pulse Width = 1 ms				Load = 100 k, Pulse Width = 5 ms				Load = 100 k, Pulse Width = 10 ms				Load = 100 k, Pulse Width = 50 ms			
		Pulses per Second = 10 PPS				Pulses per Second = 10 PPS				Pulses per Second = 10 PPS				Pulses per Second = 10 PPS			
**VoltageSetting (V)**		20	30	40	60	20	30	40	60	20	30	40	60	20	30	40	60
**Measured voltage** (V)	**Mean**	19.76	29.68	39.53	59.27	19.86	29.62	39.56	59.47	19.79	29.68	39.73	59.57	19.86	29.88	39.73	59.37
	**SD**	0.26	0.41	0.64	0.87	0.31	0.38	0.61	0.82	0.20	0.35	0.55	0.67	0.20	0.41	0.44	0.67
	**Error (%)**	2.50	2.43	2.78	2.67	2.25	2.54	2.62	2.25	2.05	2.23	2.05	1.83	1.70	1.77	1.78	2.17

**Table 2:** Error estimation of the electroporation on generating electric pulses with specific pulse width.

Parameters	Load = 100 k, Pulse Voltage = 1 ms				
	Pulses per Second = 10 PPS				
**Pulse width Setting (ms)**		1.00	5.00	10.00	50.00
**Measured pulse width (ms)**	**Mean**	1.002	5.004	10.015	50.045
	**SD**	0.007	0.049	0.065	0.089
	**Error (%)**	0.900	0.980	0.650	0.178

**Table 3:** The results (mean ± SD) of simulated accuracy of the variable current source at two different load resistors.

Program Setting Stored Inside the Microprocessor Current Strength (μA)	Resistance of Resistor (k)	Measured Parameters	
		The Current Strength of the Accuracy (μA)	The Pulse and Bipolar Current Error Timing (t > 1 μS)
**100 (dc)**	10	100 ± 0.65%	-
**300 (dc)**	10	300 ± 0.73%	-
**1100 (dc)**	10	1100 ± 1.5%	-
**300 (pulse)**	10	300 ± 0.83%	<1.1%
**300 (bipolar)**	10	300 ± 0.83%	<1.3%
**100 (dc)**	100	100 ± 0.85%	-
**300 (dc)**	100	200 ± 1.24%	-
**1.1 (dc)**	100	200 ± 1.24%	-
**100 (pulse)**	100	100 ± 0.85%	<1.2%
**100 (bipolar)**	100	100 ± 0.85%	<1.5%
